# Editorial: Augmentation of Brain Function: Facts, Fiction and Controversy

**DOI:** 10.3389/fnsys.2018.00045

**Published:** 2018-09-12

**Authors:** Mikhail A. Lebedev, Ioan Opris, Manuel F. Casanova

**Affiliations:** ^1^School of Medicine, Duke University, Durham, NC, United States; ^2^Leonard M. Miller School of Medicine, Miami, FL, United States; ^3^School of Medicine Greenville, University of South Carolina, Greenville, SC, United States

**Keywords:** neuroprosthesis, implants, microcircuits, nootropics, tDCS—transcranial direct current stimulation, TMS, Brain machine interface (BMI), neural networks

## Brain augmentation: the major trends

This research topic consists of 148 articles on various aspects of brain augmentation contributed by more than 600 authors. At the time of writing, the articles have been viewed online more than 1.3 million times and received plentiful citations in the scientific literature. The topic won the 2017 Frontiers Spotlight Award.

The topic theme, “Augmentation of brain function,” is an umbrella term for the approaches from different disciplines, aimed at the improvement of brain performance in both healthy people and patients suffering from neurological disabilities. Functions of the brain that scientists hope to augment belong to sensory, motor and cognitive domains. Brain enhancements could be achieved pharmacologically or using neurostimulation. Functional improvements can be also achieved with brain training techniques that employ modern technologies like computer games and virtual reality. Furthermore, brain performance can be augmented using brain-machine interfaces (BMIs), the pathways that connect neuronal circuits to external assistive devices, such as limb prostheses, exoskeletons, and communication aids. In addition to sending commands to external devices, BMIs can enable bidirectional communications, where artificial sensory signals are delivered to the brain while information is being decoded from neural recordings.

Even though many of the brain-augmenting ideas sound like science fiction, the topic authors feel optimistic about most of them. The overall consensus is that brain performance can be improved with artificial components, and this approach will lead to practical applications in the not-too-distant future. Many of the techniques covered in the topic, for example BMIs and noninvasive stimulation, have already experienced an explosive development. While expectations are high for the augmentation approaches, philosophers are warning about the ethical issues related to technologies that interfere with the mind, possibly in unpredictable ways. Although some of these concerns seem far-fetched, it is important that ethical standards are kept high as these revolutionary brain-augmenting methods are being developed.

The 10 most viewed articles in the topic (“1” is the highest rank) highlight the major trends in brain augmentation research:

1. *Performance enhancement at the cost of potential brain plasticity: neural ramifications of nootropic drugs in the healthy developing brain* (Urban and Gao). This a review of the main classes of drugs potentially capable of the enhancement of cognitive functions in healthy individuals. The authors warn, however, about the unwanted consequences of these pharmacological approaches, such as the development of addictive behaviors and detrimental effects related to drug overdose. These drugs could induce brain plasticity that interferes with normal brain functions and their development, particularly in young individuals.
2. “*Non-invasive” brain stimulation is not non-invasive* (Davis and Koningsbruggen). This opinion article critically evaluates the methods known as “noninvasive brain stimulation,” such as transcranial magnetic stimulation (TMS) and transcranial direct current stimulation (tDCS). Although these approaches do not require physical penetration of an instrument in the body (i.e., the medical definition of “noninvasive”), they could affect the brain in profound ways, for example by causing long-lasting plasticity that extends beyond the intended augmentation effect. Therefore, safety is an important concern, and perhaps the terminology should be changed so that non- expert users are not misled that the effects of these methods are mild.
3. *Augmentation of cognitive brain functions with transcranial lasers* (Gonzalez-Lima and Barrett). This article suggests that transcranial stimulation with infrared lasers could affect brain bioenergetics in a positive way, and that frontal cortex functions, such as attention, working memory, and affective state, could be augmented with such stimulation.
4. *Increased intelligence is a myth (so far)* (Haier). This article points to the problem of quantifying the effects of cognitive training. Haier argues that it is difficult to evaluate the enhancement of mental abilities with tests such as intelligence scores, which are prone to errors. These scores have high test-retest reliability but their standard error across subjects is high. A better estimate of intelligence could be provided with a battery of intelligence tests instead of a single test.
5. *Attitudes toward pharmacological cognitive enhancement—a review* (Schelle et al.). This article analyzes 40 published studies on public attitude toward using drugs to achieve cognitive enhancement. The authors conclude that the public concerns regarding pharmacological enhancement – medical safety, coercion, and fairness – match the agenda of academic debates.
6. *Sleep for cognitive enhancement* (Diekelmann). This article reviews the potential of sleep for augmenting such cognitive functions as attention, language, reasoning, decision making, learning and memory. The article also discusses the role of sleep in memory consolidation and the acquisition of new memories after sleep, the role of sleep-specific brain oscillations in these processes, and the neurotransmitters involved. Diekelmann describes how memory processing during sleep can be augmented by cueing memory reactivation with olfactory and auditory cues, electrically inducing sleep-specific brain oscillations, and modulating specific neurotransmitter systems pharmacologically.
7. *Transcranial direct current stimulation: five important issues we aren't discussing (but probably should be)* (Horvath et al.). This article discusses several key issues related to the usage of tDCS as a cognitive enhancement approach: (1) inter-subject variability and the need for an individualized approach, (2) intra-subject reliability, such as reliability over time, (3) the importance of proper controls in tDCS studies, such as sham stimulation and blinding, (4) interference of motor and cognitive activities with the tDCS effects, and (5) changes in electric current related to hair thickness and electrode attachment methods.
8. *Donor/recipient enhancement of memory in rat hippocampus* (Deadwyler et al.). This article reports an augmentation approach, where the memory content of one subject is transferred to the brain of another subject using electrical stimulation. The study was conducted in rats. The information was read out from the hippocampus of a “donor” rat performing a difficult long-delay behavioral task. This signal was then processed by a multiple-input multiple-output model and delivered to the hippocampus of the “recipient” animal that utilized this memory trace to reproduce the task performance.
9. *Enhancement of cognitive and neural functions through complex reasoning training: evidence from normal and clinical populations* (Chapman and Mudar). This article describes an approach, where enhancement in higher-order brain functions, such as reasoning, is achieved through cognitive training. The training includes such strategies as strategic attention, integrated reasoning, and innovation. The authors argue that cognitive training can be efficient in both healthy subjects and patients.
10. *When is diminishment a form of enhancement? Rethinking the enhancementdebate in biomedical ethics* (Earp et al.). This article discusses cases where a diminishment of certain functions could have a positive effect on an individual's well-being and thus act as a form of enhancement. For example, TMS could disrupt one brain function but by doing so enhance another function.

These and other articles in the research topic express three main ideas regarding the approaches to brain augmentation. The first is the idea of *decoding* information from brain activity. Neural signals can be sampled with various recording methods. The decoded brain signals could be processed by a BMI and utilized to augment motor, sensory, and cognitive functions. The second idea is the proposal that the brain could be augmented by *stimulation*; for example, modulating neural circuits by the application of electrical/optogenetic stimulation or using pharmacological agents to affect neural processing. The third idea is a *futuristic* vision of radical improvements of individual humans and mankind, such as revolutionary clinical approaches, immortality of consciousness, and even brain-to-brain communications. We used these themes to group the articles into three volumes. Volume I covers the approaches for recording and decoding neural signals with BMIs; Volume II is a collection of articles on neurostimulation and pharmacological methods; and Volume III describes clinical applications of brain- augmenting methods, futuristic ideas, and ethical issues.

## Volume I: brain-machine interfaces

BMIs are the major theme of Volume I. The articles cover a wide range of BMI applications, including the traditional ones and BMIs those that have emerged relatively recently. The notable new developments in this field are the BMIs for controlling bipedal walking (Wall et al.; Lisi et al.; Zarka et al.; Bouyarmane et al.; Li W. et al., Solopova et al.; Song W. et al.; Onose et al.), BMIs that modulate attention (Astrand et al.; Ordikhani-Seyedlar et al.), and technologies that enhance the human ability to predict future events (Pezzulo et al.). As any rapidly developing field of science, the BMI field is not without challenges and controversies (Vassanelli and Mahmud; Serruya; Bauer and Gharabaghi). To this end, several articles critically evaluate the current state of the field and propose improvements for the future (Waldert; Zehr; Deca and Koene; Serruya). One pressing issue is the relatively low information transfer rate (ITR) of current BMIs. Baranauskas discusses the major factors limiting the ITR and proposes that a better understanding of neural mechanisms is needed to improve BMI accuracy and versatility. Several articles describe the specific neural mechanisms that could be utilized in BMIs and other brain-augmenting approaches to improve their efficiency. Thus, Opris and Casanova highlight the need for better understanding of brain microcircuits in healthy people and neurologically impaired patients. Mandonnet and Duffau propose that the cerebral circuitry engaged in cognition and action should be thoroughly investigated for the BMI methods to be effective, and Taya et al. argue that connectome approach should be combined with cognitive enhancement methods. Furthermore, Hales and Pockett argue that a better understanding is needed of the electrical fields produced by brain circuits, and Saniotis et al. discuss evolutionary challenges related to using BMIs and other augmenting methods.

When designing brain-augmenting systems, it is important to understand the brain states targeted by these technologies. Sleep is one such state to which augmenting methods could be applied. As mentioned above, one of the articles (Diekelmann) discusses at length how sleep mechanisms could be employed for brain augmentation. Additionally, Pigarev and Pigareva highlight two sleep-related phenomena that are relevant to brain-augmenting approaches: partial sleep and visceral processing during sleep.

Action planning and decision making are the other neural functions where augmenting methods could be applied. To this end, Mirabella reviews neuronal mechanisms of goal-directed actions and links them to the research on brain augmentation. Furthermore, Opris et al. report that the relationship between neostriatal activity and movement kinematics is affected by the degree of certainty about the reward that could result from the motor act. Therefore, BMIs that decode kinematic parameters of movements should incorporate a model of reward representation. As a matter of fact, reward (or reinforcement) is an integral part of any BMI system, and operation of some of these systems is explicitly described as self-regulation of brain activity based on reinforcement learning. Yet, Wood et al. clarify that self-control of neural activity should be distinguished from the broader concept of BMI control and propose a framework that considers the interplay of automatic and controlled information processing. Moreover, Deepeshwar et al. and Telles et al. advocate meditation as a self-control paradigm for augmenting the brain.

For a brain-augmenting method to be efficient, it should properly accommodate mechanisms of brain plasticity. Several Volume I articles discuss such mechanisms. Di Pino et al. and Sakurai review brain plasticity caused by the use of artificial augmenting effectors. Sakurai et al. describe operant conditioning of neural circuits that could cause plasticity Without a change in behavior? Benyamini and Zacksenhouse present evidence showing that brain circuits act very much like an optimal feedback controller during the adaptation to BMI control. Additionally, Qi et al. report that the somatosensory system in mature primates is capable of plasticity that compensates for unilateral lesions of dorsal column afferents. Finally, Frye et al. suggest that brain-augmentation research should not be limited to the brain tissue because the peripheral organs also play a role in modulating and augmenting brain functions. In support of this suggestion, they have found a liver factor involved in neural plasticity.

BMI performance critically depends on the type of neural signal being recorded and decoded. Invasive and noninvasive BMIs are the two major classes of BMIs defined by the recording method. Waldert discusses the pros and cons of invasive and noninvasive recordings and the future of these approaches. Among the noninvasive methods, electroencephalography (EEG) is the most popular approach utilized in BMIs. Obeid and Picone report an EEG database collected by Temple University Hospital. This database could be useful for the exploration of neural representation of information and development of BMI decoding algorithms. Callan et al. assess the efficiency of dry EEG recordings; they argue that this method is useful for decoding of auditory events from EEG data, even when substantial acoustic noise, and mechanical and physiological artifacts, interfere with the recordings during simulated and real flight conditions. Blankertz at al. review different usages of EEG-based BMIs, including practically oriented applications and employing BMIs as research tools.

While EEG-based BMIs are easy to implement and safe to use, their information transfer rate (ITR) is limited. ITR can be improved if electrical activity is recorded from the surface of the brain using electrocorticography (ECoG), a minimally invasive method. Kapeller et al. report a high-performing ECoG BMI based on visual evoked potentials, and Zippo et al. describe a novel epicortical grid with wireless recordings, tested in rhesus monkeys.

Functional magnetic resonance imaging is the other noninvasive recording method suitable for BMI implementations. Caria discusses the neurophysiological mechanisms involved in self-regulation of blood oxygenation level monitored with fMRI.

Intracranial recordings hold the promise of radically improving the quality of neural signals utilized in BMIs. However, this potential has not yet been realized because of safety and longevity issues with brain implants. Technologies are developing rapidly for making invasive implants more efficient. Among such technologies, nanostructure-based recording sensors are particularly promising (Vidu et al.). Additionally, Agorelius et al. describe a new multichannel implant composed of flexible electrodes to minimize damage to the brain tissue.

In addition to “pure” BMIs (i.e., the ones based on the recordings from the brain only) additional types of bioelectric recordings may be useful for brain-augmenting methods. Shishkin et al. report a hybrid BMI that, in addition to extracting motor intentions from EEG activity, utilizes recordings of eye position EEG markers of gaze fixation to improve BMI performance. Interfaces based on electromyographic (EMG) recordings are another class of augmenting devices that could operate a myoelectric hand prostheses for amputees (Atzori and Müller) or convert forearm electromyographic (EMG) activity into traces for handwriting (Okorokova et al.; Okorokova et al.).

The choice of sensory feedback is another important factor that fundamentally affects BMI performance. Several articles investigate different types of feedback. Alimardani et al. report manipulations with visual feedback that improve the operation of motor-imagery BMIs. BMI performance can be further improved using multisensory stimuli as the feedback (Thurlings et al.), such as combining visual feedback with haptic (Honeine and Schieppati; Bouchard et al.) and auditory (Tonelli et al.) inputs. Oei and Patterson describe the feedback provided by action videogames; they show that videogames have similar demands as many other perceptual and attention tasks, which explains transfer of functional enhancements resulting from playing these games. Wright examines the visual feedback provided by virtual reality; the article discusses how virtual reality could be used as a brain-augmenting approach. Alfaro et al. report the results of a neuroimaging study, in which they examined plasticity in visual and auditory areas of a color-blind subject with eight years of training to utilize a device called “Eyeborg” that transforms colors into sounds. Finally, Bravi et al. report improvements of somatosensory feedback with elastic therapeutic tape.

BMI performance can be improved with better decoding algorithms. The article by Li reviews the current state of research in this field. Rouse and Schieber argue that BMI decoders should incorporate non-linear characteristics to advance BMIs to better match natural motor performance. Lebedev discusses how different BMI decoders could be assessed using neuron-dropping curves.

## Volume II: neurostimulation and pharmacological approaches

In Volume II, one group of articles covers a variety of neurostimulation methods (Balan et al.), while the other group describes pharmacological approaches. Electrical stimulation is a conventional method for inducing brain activity. The types of electrical stimulation include intracortical microstimulation (ICMS), transcranial direct current stimulation (tDCS), and transcranial magnetic stimulation (TMS) that induces electrical currents in the nervous tissue. Electrical stimulation can be also be applied to muscles (Talis et al.).

Noninvasive stimulation methods have gained popularity in recent years as a means of augmenting brain function, yet many unknowns and controversies still remain. Krause and Cohen Kadosh and Horvath et al. examine the role of inter-subject differences in responsiveness to tDCS. McKendrick et al. propose an approach in which wearable devices are used that combine tDCS with a new generation of miniaturized fNIRS systems. Blumberg et al. and Foroughi et al. report that performance on spatial tasks can be enhanced by tDCS applied to the posterior parietal cortex. Younger et al. demonstrate that tDCS applied to the left inferior parietal lobe can augment reading subskills. Luft et al. propose that a connectome approach can be combined with brain stimulation. Horschig et al. discuss neurostimulation methods that could be used to manipulate cortical oscillations. Koganemaru et al. and Tsagaris et al. suggest that the efficiency of neurostimulation can be improved if it is combined with the appropriate task patterns.

As mentioned above, Davis and Koningsbruggen do not think that the term “noninvasive” is appropriate to describe noninvasive stimulation methods that strongly affect the brain and evoke long- lasting consequences. Therefore, these approaches should be used with great caution. Among the effects of noninvasive stimulation the authors of the topic name biasing network dynamics (Wokke et al.) and influencing brain hemodynamics (Pulgar; Dutta). Additionally, brain functions can be affected even with transcranial lasers (Gonzalez-Lima and Barrett). Duecker et al. discuss the potential of noninvasive stimulation as a research tool in the studies of perception, cognition, and behavior. Additionally, Luber argues that brain augmentation with noninvasive stimulation cannot be explained by a net zero sum proposition; i.e., the mechanism where brain resources are reallocated: gains in one function are balanced by costs elsewhere.

Among the invasive approaches to neurostimulation, optogenetic methods have received particular attention in recent years, and several articles in Volume II cover different aspects of this approach (Kwon et al., Jarvis and Schultz). While the optogenetic methods are being developed, the classical electrical stimulation approach has already resulted in clinically relevant applications like the vestibular implant (van der Berg et al.) and tactile neuroprosthesis that utilizes intracortical microstimulation (Kim et al.).

Memory prostheses represent a recent trend in stimulation-based BMIs. Volume II covers several memory-augmenting approaches. Madan, Bennabi et al., Deveau et al., Moreau, Mallow et al., and Takeuchi et al. discuss different ways to implement neuroprosthetic memory. Song D. et al. report a memory prosthesis, where memory content is extracted from hippocampal activity using a multiple-input, multiple-output non-linear dynamical model. In addition to neurostimulation, pharmacological approaches have been developed for augmenting memory and cognition (Lynch et al.). Brain training techniques can be also used to improve memory and cognition (Chapman and Mudar; Ben-Soussan et al.; Haier). In particular, Beatty et al. investigate several working memory tasks where training could be transferred from one task to another. Additionally, Rabinovich et al. propose a computational model to explore the ways of augmenting memory. The model is based on recurrent inhibitory-excitatory networks with heterogeneous inhibition.

As far as pharmacological approaches to brain augmentation are concerned, Urban and Gao and Lynch et al. discuss the pros and cons of using nootropic drugs to augment brain performance. The concern regarding nootropic drugs is significant and is shared by general public (Schelle, Faulmüller et al.; Schelle, Olthof, et al.; Garasic and Lavazza). Using an individual-oriented approach, Jellen et al. propose a method for screening and personalizing nootropic drugs; the method utilizes gene expression data to evaluate affected signaling pathways. Piermacino et al. review the effects of pharmacological interventions on cognition in the elderly, while Kang et al. review the effects of acetylcholine in the primary visual cortex that could be used to alter and augment visual perception.

## Volume III: from clinical applications to ethical issues and futuristic ideas

Developing clinical applications is perhaps the most important direction of the brain- augmentation field (Schicktanz et al.), and many Volume III articles demonstrate the progress that has been achieved already in clinical solutions for such conditions as epilepsy (Höller and Trinka; DeMarse and Carney; Zeitler and Tass), stroke (Grimm et al.), Parkinson's disease (Lebedev et al.; Lee et al.), Huntington's disease (Nagel et al.), dementia (Garriga et al.; Franco), Alzheimer's disease (Yegla and Parikh), autism spectral disorders (ASD) (Billeci et al.), traumatic brain injury (Alwis and Rajan; Tajiri et al.), and disorders of consciousness (Bai et al.). Evidence is growing that noninvasive stimulation can be employed to treat a range of neurological conditions (Vicario and Nitsche). Thus, Sokhadze et al. report that TMS applied to dorso-lateral prefrontal cortex improves executive function in ASD, which is evident from the improvements in behavioral reactions and event-related EEG potentials. Additionally, according to the case report by Brem et al., tDCS can be applied to treat visuospatial neglect. Krawinkel et al. provide insights on how noninvasive stimulation could treat such conditions as schizophrenia and Parkinson's disease by modulating brain oscillations. Additionally, Kubera et al. review the application of noninvasive brain stimulation to the treatment of auditory verbal hallucinations in schizophrenia. Moreover, Ayache et al. report that prefrontal tDCS can decrease pain in patients suffering from multiple sclerosis. Charvet et al. propose that therapeutic noninvasive stimulation can be administered remotely under the supervision of medical personnel, which removes the need for the patients to travel to the hospital, and Thibeault argues that efficiency of therapeutic neurostimulation can be improved by neuromorphic components.

The articles that we call futuristic examine the prospects for augmentation methods that only recently migrated from science fiction to scientific theory and research. Thus, Kennedy argues that advances in BMI technologies could help mankind cope with the “moment of singularity”, the time when artificial intelligence surpasses human intelligence. Brain-to-brain interfaces that enable communications between several individual brains are another futuristic idea implemented in several studies (Hildt). For example, Deadwyler et al. demonstrate that brain-to-brain communications can be employed to transfer memories (see also the summary of this article above). Kyriazis extends the idea of brain-to-brain interface even further by proposing a global brain, a self-organizing system that connects many humans. Additionally, Sexton and Lukinova and Myagkov, discuss the role of social interactions in the operations of augmenting technologies. Finally, augmenting methods could be applied to modulate consciousness (Berry and Parker), even though its neural mechanisms are poorly understood (Pockett).

The topic attracted a considerable number of the articles on ethical issues related to the brain- augmenting methods (Glannon; Glannon; Attiah and Farah; Nagel; Clark; Maslen et al.), including the relationship between the diminishment and enhancement following the application of brain-augmenting technologies (Earp et al.), the problem of “mind control” with BMI technologies (Koivuniemi and Otto), free will (Glannon; Glannon), the duty to use cognitive enhancers in high-responsibility professions (Santoni et al.), determining the population of people in need of brain enhancement (Schleim), informed public policy (Shook et al.), cognitive biases (Caviola et al.), and the hype caused by the development of brain- augmenting approaches (Rusconi and Mitchener-Nissen).

## Author contributions

All authors listed have made a substantial, direct and intellectual contribution to the work, and approved it for publication.

### Conflict of interest statement

The authors declare that the research was conducted in the absence of any commercial or financial relationships that could be construed as a potential conflict of interest.

